# The impact of adding orange peel waste on the engineering proprieties of fired clay bricks

**DOI:** 10.1038/s41598-026-69584-2

**Published:** 2026-09-15

**Authors:** M. M. Ahmed, Ahmed M. Ebid

**Affiliations:** 1 Department of Physics and Mathematics Engineering, Faculty of Engineering at Mataria, Capital University (formally Helwan University), Cairo, 11718 Egypt; 2 Structural Engineering & Construction Management Department, Future University in Egypt, Cairo, 11835 Egypt

**Keywords:** Agro-waste valorization, Fired clay bricks, Orange peel waste, Pore-forming agent, Thermal conductivity, Sustainable construction materials, Energy science and technology, Engineering, Environmental sciences, Materials science

## Abstract

The construction industry urgently requires sustainable alternatives to conventional building materials to reduce resource depletion and greenhouse gas emissions. This study systematically investigates the incorporation of orange peel waste (OPW) as an eco-friendly pore-forming agent in fired clay bricks. Brick formulations were prepared by replacing clay with OPW at dosages of 0, 2.5, 5.0, 7.5, and 10.0%, and sintered at peak firing temperatures of 700 °C, 800 °C, and 900 °C. The physical, mechanical, and thermal properties were rigorously evaluated. The results demonstrate that increasing OPW content leads to a progressive reduction in bulk density alongside an increase in apparent porosity and water absorption due to organic matter combustion. Although the induced macro-pores decreased compressive strength, specimens containing 5.0–10% OPW fired at 900 °C safely exceeded international regulatory thresholds for non-load-bearing structural applications. Furthermore, the optimized bricks exhibited a substantial drop in thermal conductivity (down to 0.42 W/m K at 10% (OPW). Ultimately, a 5.0–10% OPW incorporation fired at 900 °C offers an optimal balance between structural integrity and enhanced insulation.

## Introduction

The construction industry remains one of the largest consumers of natural resources and a significant contributor to global environmental degradation. Bricks, as fundamental building materials, have been utilized for millennia, yet their conventional production raises serious sustainability concerns^1–3^. The extensive extraction of clay for brick manufacturing has led to the depletion of natural clay deposits, while the firing process consumes substantial energy and emits considerable amounts of carbon dioxide^3,4^. Approximately 40% of all CO_2_ emissions originate from building construction and operation, with 15% directly associated with the production of construction materials^4^. These environmental challenges have prompted researchers and industry stakeholders to seek sustainable alternatives that reduce reliance on virgin raw materials and minimize ecological footprints^3,5^. In parallel, the agricultural sector generates vast quantities of biomass waste globally, posing significant disposal and environmental problems^4,6^. Crop residues, fruit peels, seeds, and other agro-industrial by-products are often burned or landfilled, contributing to air pollution, greenhouse gas emissions, and soil contamination^1,3,7^. The valorization of these agricultural wastes as alternative raw materials in brick production presents a dual opportunity: mitigating waste disposal issues while conserving natural clay resources^1,2,6^. This circular economy approach has gained considerable traction, with numerous studies investigating the incorporation of various agro-wastes into fired clay bricks^3,5^.

The addition of organic and biomass-based additives to clay bricks fundamentally alters their physical, mechanical, and thermal properties through the formation of pores during combustion^5,8,9^. Generally, the incorporation of agricultural residues reduces bulk density and thermal conductivity, producing lighter bricks with enhanced insulation capabilities^5,9,10^. However, these benefits are often accompanied by decreased compressive strength and increased water absorption and porosity^8,9,11^. The extent of these changes depends critically on the type, morphology, and percentage of the additive, as well as the firing temperature and duration^5,9,12^. Therefore, identifying the optimal additive type and concentration is essential to balance improved thermal and acoustic performance with acceptable mechanical integrity^8,13,14^.

Numerous agricultural wastes have been successfully investigated as partial clay substitutes. Potato peel powder, for instance, has been shown to reduce compressive strength by up to 88% while increasing porosity and sound insulation when added at 7% by weight^10^. Similarly, pomegranate peel waste, a widely studied biomass residue, has demonstrated significant reductions in thermal conductivity (up to 62–97.7%) and bulk density (up to 26%) at optimal concentrations of 10–15% and firing temperatures of 900–1000 °C^11,15,16^. These pomegranate peel-enhanced bricks also reduced energy consumption by 13.89% and CO_2_ emissions by 12% while offering economic payback periods as short as 0.65 years^16^. Other fruit-derived wastes, including grape seeds, cherries seeds, and wine lees, have been incorporated at 5 wt% to achieve weight reductions of 3–13% while maintaining acceptable flexural strength (21–23 MPa)^8,14^.

Citrus-based wastes have received particular attention due to their abundance and favorable characteristics. Orange peels, representing 45–50% of the total fruit mass, are rich in protein (7.15%) and crude fiber (12.79%)^17^. Arshad^18^ demonstrated that orange peels combined with coconut waste could reduce clay content by 10–60% while producing lightweight, shock-absorbing bricks that meet compressive strength requirements per ASTM and BIS standards. The porous and fibrous structure of such waste materials, as confirmed by SEM analysis, contributes to the formation of internal pores upon combustion^18^. More recently, sweet orange peels have been characterized for their potential in sustainable material applications, though their direct incorporation into fired bricks remains underexplored compared to pomegranate and potato wastes^17^.

Beyond fruit wastes, other biomass residues have been extensively evaluated. Olive pomace bottom ash has been incorporated at 10–20 wt%, yielding bricks with compressive strengths of 33.9 and 14.2 MPa, respectively, and reducing thermal conductivity by 14.4–16.8%^13^. Similarly, olive pomace and its bottom ash have been combined with coal waste, with thermal conductivity maintained below 0.4 W/m K when sintered at elevated temperatures^12^. Tea waste, sawdust, and wheat straw added at 2–6 wt% have produced bricks meeting Pakistan Building Code standards when fired at 980 °C, with notable improvements in thermo-physical properties^9^. Rice husk ash and sugarcane bagasse ash have been incorporated at 2–8%, achieving compressive strength enhancements of up to 31% and 27%, respectively, depending on clay source^19^. Wine industry wastes, including stalks, grape seeds, and wine lees, have produced lightweight bricks with density reductions up to 13% and improved thermal insulation suitable for partition walls^14^. Mushroom waste, a novel additive, reduced thermal conductivity by 62% and bulk density by 26% at 15% incorporation while maintaining compressive strength above structural requirements^20^. Sawdust and other woody biomass have been limited to approximately 10 wt% to balance positive effects (weight and shrinkage reduction, porosity increase) against negative outcomes (water absorption increase, mechanical resistance decrease)^8^.

The acoustic properties of agro-waste-enhanced bricks represent a particularly promising yet less frequently reported benefit. Ghorbani et al.^10^ reported that potato peel powder at 5% increased sound insulation by 18.62% at 5000 Hz compared to ordinary clay bricks, while sour orange leaf powder at 7% yielded a 13.68% increase in sound insulation. This finding underscores the potential of biomass additives, particularly citrus-derived materials, to improve the acoustic performance of building envelopes. The combination of enhanced thermal insulation, reduced weight, and improved acoustic properties makes such bricks highly suitable for partition walls where load-bearing requirements are less stringent and cost-effectiveness is prioritized^10,14^.

Despite the extensive body of research on various agricultural wastes in fired clay brick production, including potato peel^10,21^, pomegranate peel^11,15,16,22^, orange peel^17,18^, olive residues^12,13^, and numerous others^2,6–9,14,20,23–27^, a critical research gap remains. The specific impact of sour orange leaf (Citrus aurantium leaf) as an additive in fired clay bricks has not been systematically investigated. While Ghorbani et al.^10^ briefly compared the effects of potato peel powder to those of sour orange leaf powder, their study focused primarily on potato peel, with sour orange leaf serving only as a comparative reference rather than as the primary subject of investigation. To date, no comprehensive study has systematically examined the effects of varying sour orange leaf concentrations on the full spectrum of brick properties, including mechanical strength, physical characteristics (density, porosity, water absorption, and shrinkage), thermal conductivity, and acoustic insulation. Furthermore, the optimal percentage of sour orange leaf for balancing these competing properties has not been established, nor have the microstructural mechanisms underlying its effects been fully elucidated.

The sintering and vitrification behavior of biomass-enhanced clay bricks has been systematically studied for various agricultural residues. Srisuwan et al.^28^ demonstrated that wood ash addition (0–16 wt%) increased porosity and water absorption while reducing bulk density, with 4% wood ash fired at 1000–1100 °C achieving the ASTM minimum compressive strength requirement of 17.2 MPa. Similarly, Phonphuak^29^ reported that dry grass incorporation (0–7.5 wt%) reduced bulk density and compressive strength, with 2.5% dry grass fired at 1100 °C yielding 11.6 MPa compressive strength and 1.78 g/cm^3^ bulk density. These studies underscore the critical interplay between organic additive content, firing temperature, and vitrification—the glassy phase formation essential for consolidating the porous structure while maintaining mechanical integrity^28,29^. This mechanistic framework provides valuable context for evaluating other biomass additives, including orange peel waste.

### Novelty and objectives

The novelty and innovation of the present research lie in providing the first dedicated, systematic investigation into the use of Orange Peel Waste (OPW) as a sustainable additive in fired clay brick production. Unlike previous studies that were concerned in clay bricks treated with sour orange leaf^10^, this research is concerned in clay bricks treated with (OPW), comprehensively evaluating its potential as an eco-friendly pore-forming agent and property modifier. The unique chemical composition and fibrous structure of (OPW), which differs significantly from sour orange leaf powder and other biomass residues, may yield distinct property profiles that could advantageously balance thermal insulation, acoustic performance, and mechanical integrity.

The primary objectives of this study are: (1) to investigate the effects of incorporating different percentages of (OPW) (ranging from 0% to 10% by weight) on the physical properties (bulk density, apparent porosity, water absorption, linear shrinkage) of fired clay bricks; (2) to evaluate the mechanical properties, particularly compressive strength, of (OPW)-enhanced bricks in comparison to conventional clay bricks; (3) to assess the thermal conductivity and specific heat characteristics of the developed bricks; (4) to determine the optimal (OPW) content that achieves the best balance between lightweight properties, thermal performance, and structural integrity. By achieving these objectives, this research aims to contribute a viable, sustainable alternative brick material that simultaneously addresses agricultural waste management challenges and the construction industry’s need for environmentally friendly building products.

## Methodology

### Raw materials

In this research, locally sourced clay was utilized as the primary raw material for the fabrication of the eco-friendly fired bricks. The primary additive evaluated was orange peel waste (OPW), which was sourced from domestic refuse and urban kitchens in Cairo, Egypt, during the winter season. To prepare the organic residue for incorporation, the raw peels underwent a rigorous washing protocol involving 4–5 cycles with tap water to effectively eliminate adhering dirt, superficial impurities, and residual soluble sugars. Following washing, the peels were chopped into small fragments and left under ambient laboratory conditions to undergo preliminary moisture loss. Final dehydration was executed in a low-temperature drying oven at 50 °C for 24 h to achieve thermal stability while preserving the intrinsic organic structure of the material.

**Fig. 1:**
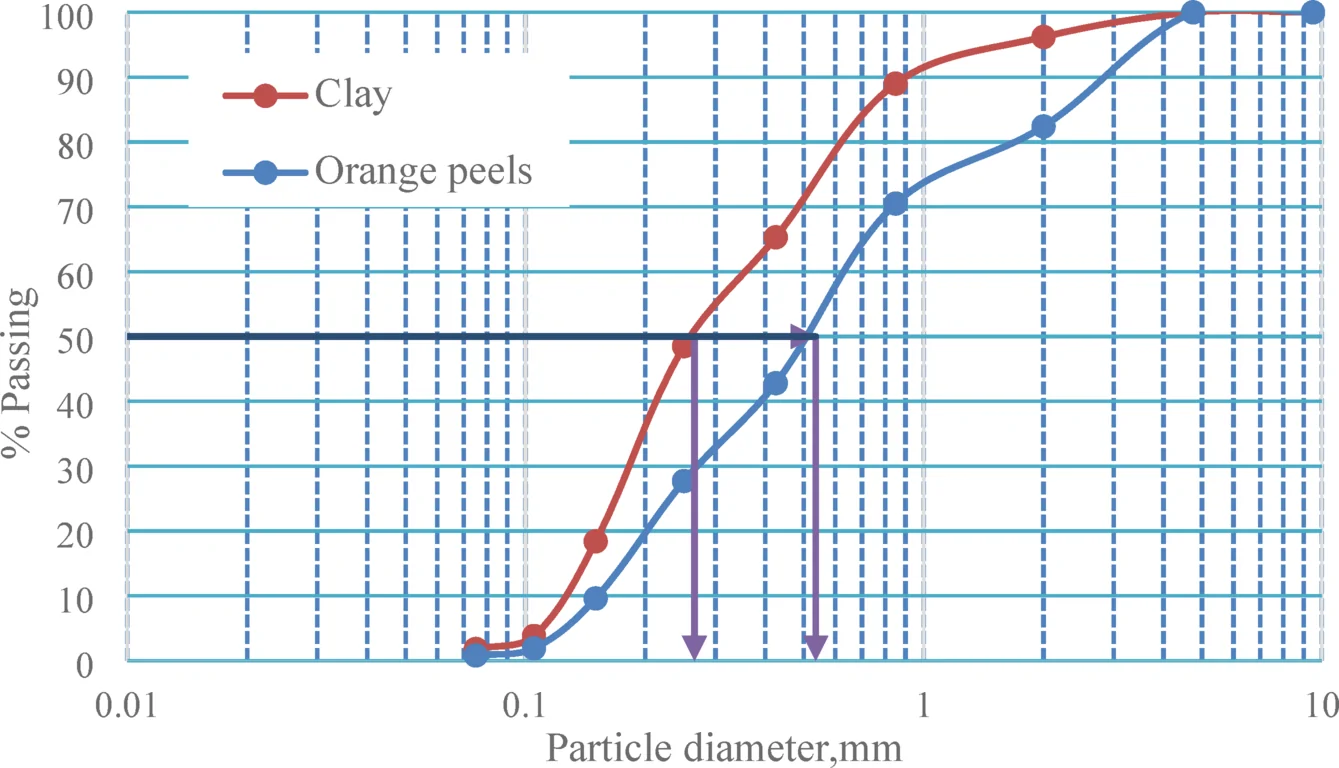
Grain size analysis of clay and orange peels.

The fully dried orange peels were mechanically pulverized into a fine powder using an electric blender and a ball mill. The resulting powder was sieved to yield fine particles with a highly uniform size distribution and homogeneous morphology, a step critical for ensuring reproducible mixing kinetics and an even distribution within the silicate clay matrix. Figure 1 shows the particle size analysis of kaolin clay and orange peels, the average diameter was 0.25 and 0.55 mm, respectively.

**Fig. 2:**
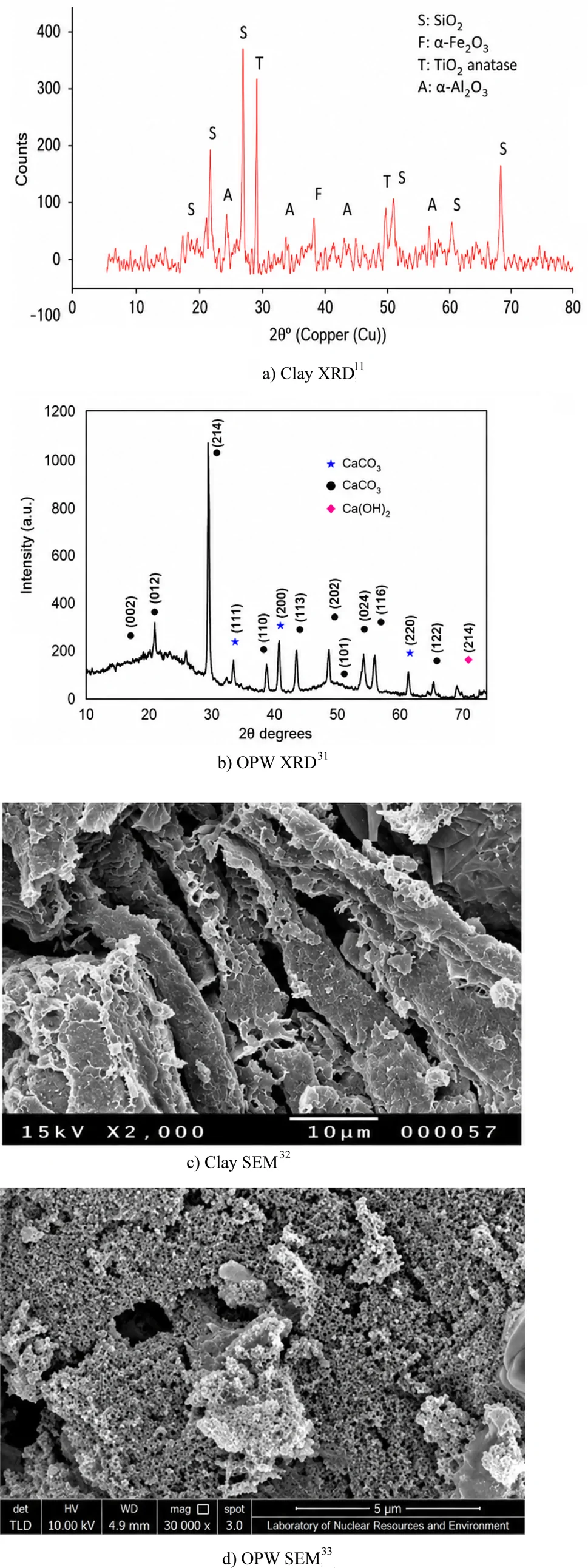
Typical XRD, (**a**) for clay (**b**) for OPW and SEM (**c**) for clay (**d**) for OPW

To characterize the physical and chemical properties of the precursor materials, a suite of analytical techniques was deployed:

- Chemical composition: The elemental oxide configurations of both the raw clay and the processed OPW were quantitatively determined via X-ray fluorescence (XRF) spectroscopy (Table 1). The loss on ignition (LOI) was monitored to quantify organic content and metal carbonate decomposition. The results reveal that OPW exhibits a significantly higher loss on ignition (LOI, wt%) compared to clay, reflecting its elevated organic content. This high LOI is attributed to the presence of organic matter and minor inorganic constituents, such as carbonates and sulfates, which undergo thermal decomposition and release volatile components (e.g., H_2_O and CO_2_) during calcination. Although silicon is a key constituent in brick manufacturing, the recommended SiO_2_ content for conventional fired bricks typically ranges between 20 and 50 wt% as reported by H. Kurmus^30^.
- Mineralogical phase identification: X-ray diffraction (XRD) analysis was performed on both the clay and the OPW powder to evaluate the dominant mineral phases and crystalline structures. See Fig. 2.
- Particle size distribution: Standard particle size analysis techniques were implemented to establish the average particle diameters of the constituent powders, optimizing matrix compatibility and dispersion uniformity.
- Microstructural and elemental mapping: To investigate the localized microstructural morphology and elemental distribution within the composite matrices, Scanning Electron Microscopy (SEM) and Energy-Dispersive X-ray Spectroscopy (EDX) were performed on selected fractured brick specimens containing 2.5 wt% and 10 wt% OPW.

Prior to batching, the processed OPW powder was sealed in low-density polyethylene (LDPE) bags and preserved in a frost-free freezer at − 22 °C to inhibit potential biological degradation and hygroscopic moisture uptake.

**Table 1:** XRF of l.

Oxides composition	Clay Wt. (%)	OPW Wt. (%)
Al_2_O_3_	30.906	0.02
SiO_2_	48.931	0.09
Na_2_O	1.097	0.08
K_2_O	1.011	0.19
CaO	0.905	2.1
MgO	0.09	0.14
TiO_2_	5.518	ND
Fe_2_O_3_	1.193	0.15
SO_3_	0.291	0.16
P2O5	ND	0.04
Cl	0.011	ND
LOI	9.58	96.65
Total	99.53	99.62

### Mix design and specimen preparation

**Fig. 3:**
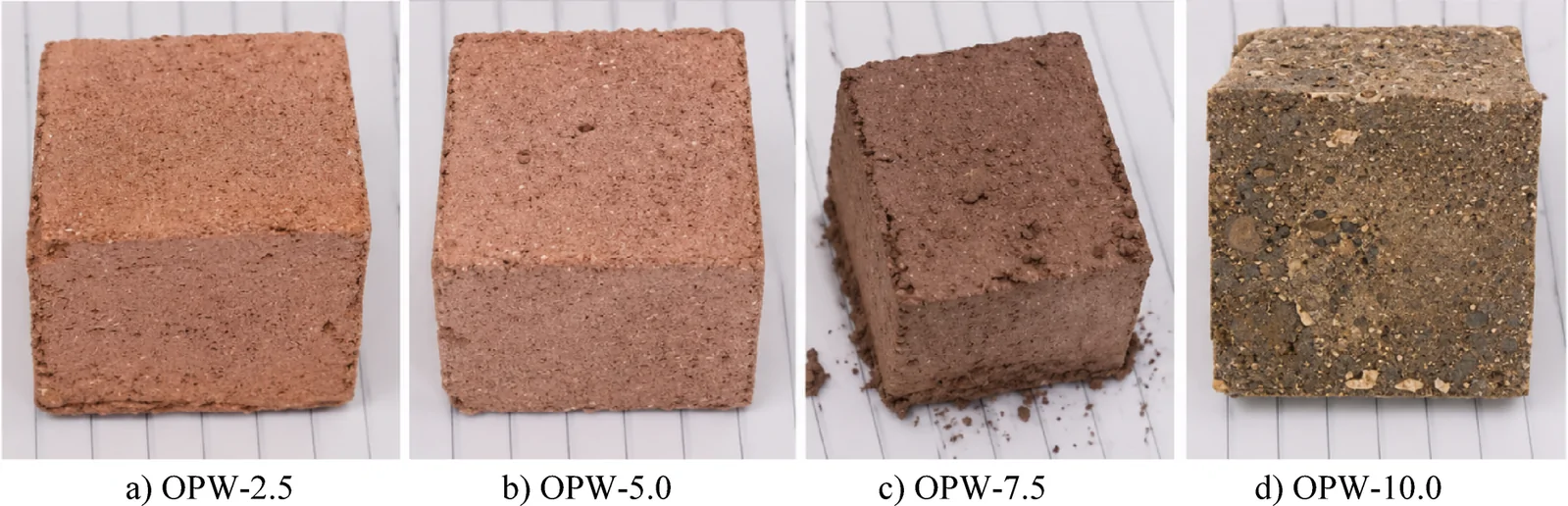
Typical photos for brick samples at different OPW doses.

A systematic experimental program was established to investigate the textural, physical, and physic-mechanical alterations induced by the organic additive. A total of five distinct brick formulations were engineered using a semi-dry pressing process. The control mix, designated as OPW-0, consisted entirely of raw clay paste without organic fractions. The remaining four mixtures incorporated OPW as a partial mass replacement for clay at dosages of 2.5, 5.0, 7.5, and 10.0 wt%, labeled as OPW-2.5, OPW-5, OPW-7.5, and OPW-10, respectively. To establish statistical reliability and ensure consistent experimental findings, 21 replicate brick specimens were fabricated for each distinct mix design. Figure 3 shows some dried brick samples.

The selection of 10% as the maximum dosage was based on several considerations:

- Prior literature: Previous studies on similar agricultural wastes have shown that exceeding 10–15% typically leads to unacceptable mechanical properties. For potato peel powder, strengths decreased by up to 88% at 7%. For pomegranate peel waste, the optimal range was identified as 10–15%. For sawdust and woody biomass, the recommended limit is approximately 10% to maintain structural integrity.
- At OPW contents above 10%, the mixture becomes increasingly difficult to mold and compact due to the reduction in clay plasticity. The high water demand of organic particles (observed as increasing water content from 18% to 34% as OPW increased from 0% to 10%) would become problematic at higher dosages.
- The 10% dosage already produced compressive strengths approaching the minimum regulatory thresholds. Higher dosages would likely render the bricks unsuitable for even non-load-bearing applications.
- Linear firing shrinkage at 10% OPW reached 7.07% at 900 °C, which is near the practical limit of 10% beyond which excessive warping and cracking would occur.

The water content was determined using Pfeffrkorn plasticity which is used to measure the plasticity of clay. The water content was adjusted to achieve adequate workability, increasing proportionally with the OPW replacement ratio. The formulations with 2.5%, 5%, 7.5%, and 10% OPW required approximately 18%, 25%, 29%, and 34% water, respectively.

The step-by-step fabrication sequence proceeded as follows:

- Pre-drying and dry mixing: The raw clay was thermally dehydrated in a laboratory oven at 100 °C for 6 h to eliminate baseline moisture. The dry clay and corresponding mass percentages of OPW powder were loaded into a custom-designed mechanical mixer featuring two steel counter-rotating axes. The dry components were blended at a rotational speed of 120 rpm using an electric stirrer for 15 min to guarantee absolute homogeneity.
- Hydration and plasticizing: Distilled water was incrementally introduced into the dry blends. The baseline water content started at 18 wt% for the control mix and was progressively adjusted upward across the series (ranging between 18 and 34 ml total volume) as the OPW percentage increased. The addition of water ensures adequate plasticity desirable for molding.
- This upward scaling was required to compensate for the higher water demand of the organic particles and to optimize the plasticity of the molding paste.
- Molding and compaction: The prepared plastic pastes were placed into standard 50 mm × 50 mm × 50 mm cubic steel molds. To minimize the occurrence of entrapped air pockets and internal macro-voids, the mixture was cast sequentially in three distinct layers. Each layer was subjected to 10 manual strokes using a 1-cm diameter metal tamping rod, followed by a 30-second consolidation cycle on a laboratory vibration table. The molded specimens were subsequently compressed under a uniaxial hydraulic pressure of 10 MPa.
- Drying and sintering: The green brick cubes were remolded and allowed to be cured at ambient laboratory conditions for 24 h, followed by a secondary drying phase in a ventilated oven at 100–120 °C for 12 h to mitigate macro-cracking risks and optimize green compressive strength. The dried specimens were then transferred into a programmable muffle furnace for high-temperature sintering. Separate batches were heated to three target peak firing temperatures: 700 °C, 800 °C, and 900 °C. A strict, conservative heating rate of 5 °C/min to 10 °C/min was enforced, and the peak temperatures were maintained for a soaking period of 4 h. This slow thermal ramp was critical to control the outgassing kinetics of volatile organic constituents and prevent internal gas entrapment, structural bloating, or micro-fissuring. Finally, the furnace was deactivated, and the sintered bricks were allowed to cool slowly to room temperature via natural convection over a 24-hour period before testing.

**Fig. 4:**
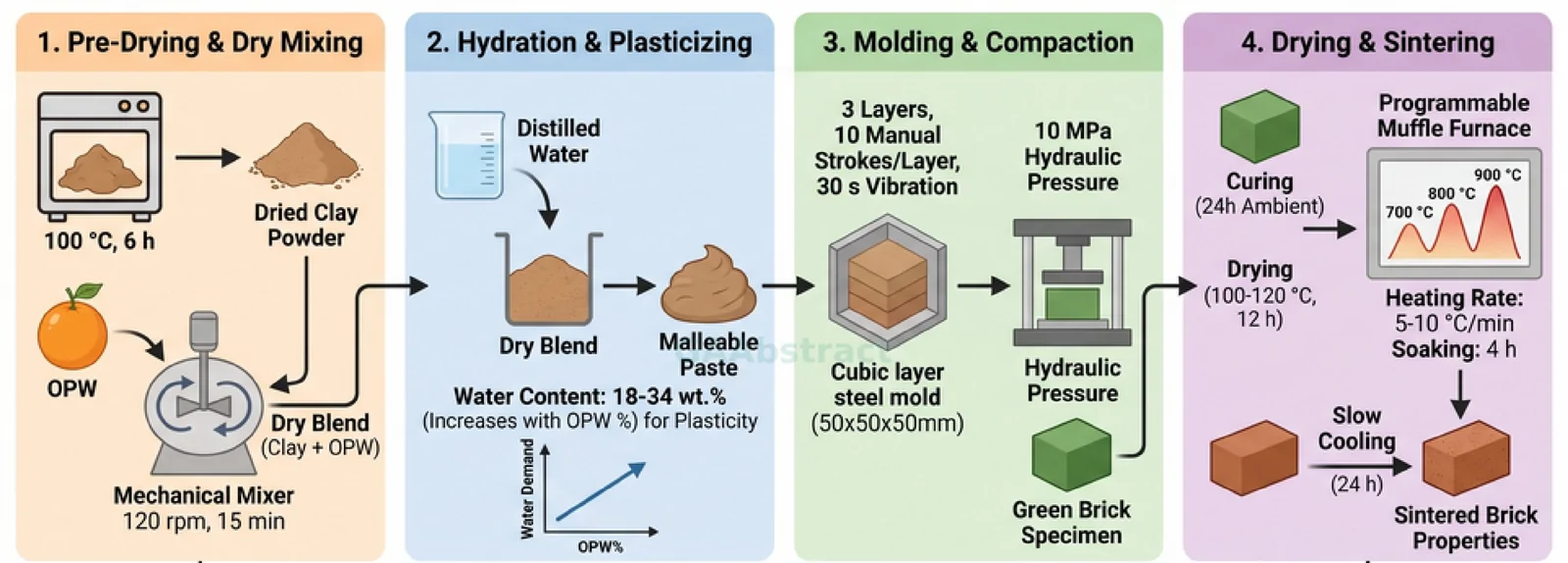
Sequence of samples fabrication.

The described procedure is graphically presented in Fig. 4.

### Testing procedures

**Fig. 5:**
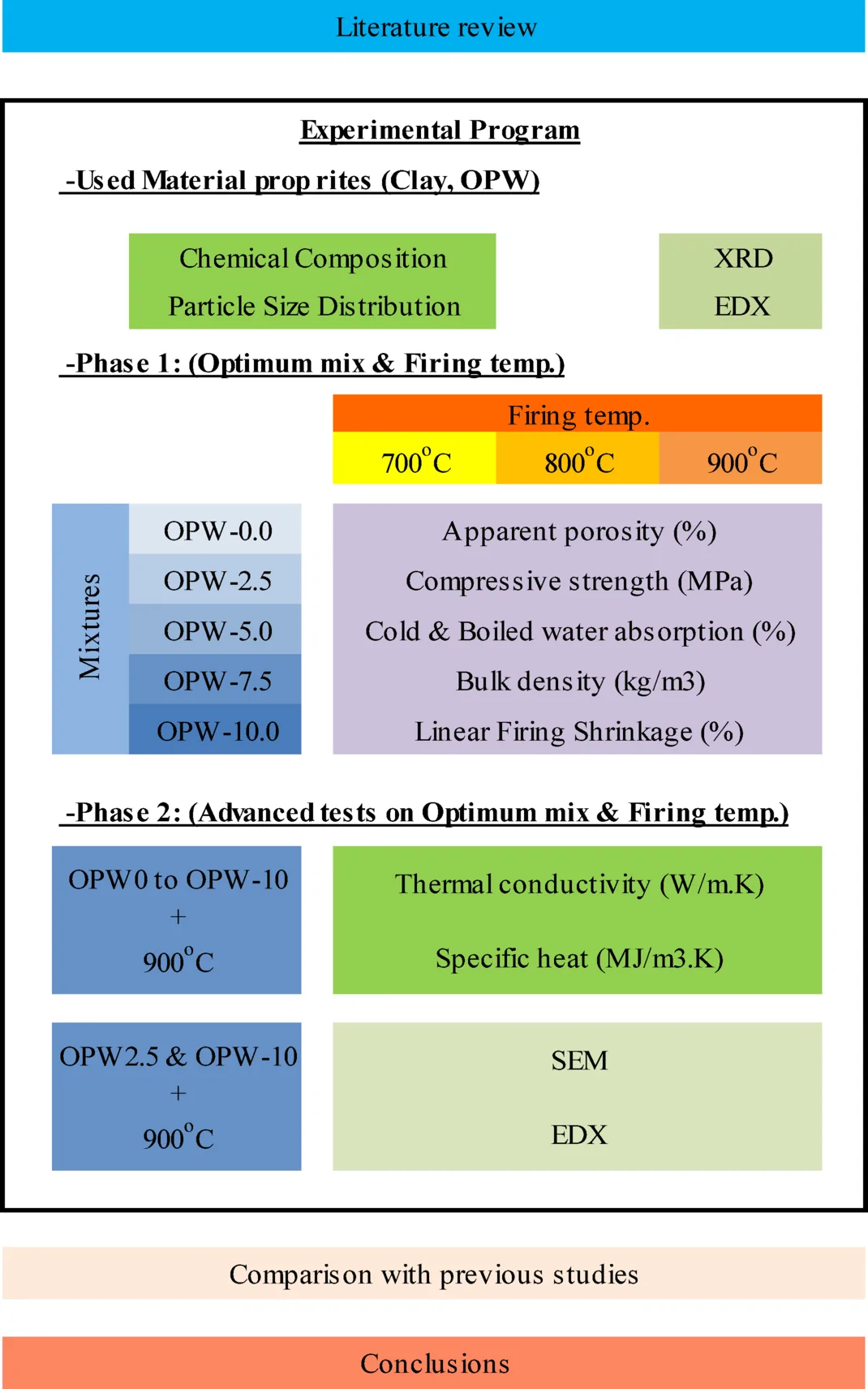
The considered research methodology.

The physical, mechanical, and thermal properties of both the green and fired brick specimens were rigorously quantified using standardized testing protocols. For every individual property evaluation, three replicate specimens were tested per batch, and the final reported parameter represents the arithmetic mean of the recorded data. In order to minimize the costs of this self-funded research, the considered methodology (as shown in Fig. 5) has two phases, the first one aims to determine the optimum mixing ratio and firing temperature that leads to higher strength, lower water absorption and lower linear shrinkage. To achieve this goal, low-cost tests (compressive strength tests, absorption tests, and linear shrinkage tests) were conducted on samples with different mixing ratios and different firing temperatures. The second phase aims to conduct advanced and high-cost tests on selected mixture at the optimum firing temperature to investigate their thermal characteristics, microstructures, elemental composition and mineralogical properties.

#### Linear drying shrinkage (LDS) and linear firing shrinkage (LFS)

Dimensional stability during the processing phases was assessed using a digital vernier caliper with an accuracy of 0.05 mm. The Linear Drying Shrinkage (LDS) was determined by measuring the change in nominal length from the green plastic state to the oven-dried state according to ASTM C326 guidelines^34^. The LDS percentage was computed via Eq. (1):

1 $$ {\mathrm{LDS}}\% = 100 \times \left( {{\mathrm{L}}_{{\mathrm{f}}} - {\mathrm{L}}_{{\mathrm{d}}} } \right)/{\mathrm{L}}_{{\mathrm{p}}} $$

where L_f_ represents the initial plastic length of the green specimen (mm) and L_d_ represents the dry length after oven processing (mm).

The Linear Firing Shrinkage (LFS) was evaluated to isolate the dimensional contractions occurring purely due to high-temperature mineral sintering and organic burnout. Flat test specimens were cast, dried to a constant weight, fired at their respective target temperatures (700 °C, 800 °C, and 900 °C) for 4 h, and checked for post-firing length variations.

#### Bulk density, apparent porosity, and water absorption (cold and boiled)

Physical configurations including bulk density, apparent porosity, and moisture kinetics were evaluated in strict compliance with ASTM C 373-18^35^ standards. The fired specimens were subjected to a 5-hour immersion protocol in a boiling water bath and subsequently allowed to cool naturally to room temperature to ensure complete saturation of all open pore networks. Upon removal, surface moisture was quickly blotted using a damp, soft cloth, and the saturated mass was recorded.

The physical parameters were calculated using the following mathematical expressions:

- Water absorption (WA, %): Measures the capacity of the open capillary network to retain water as per Eq. (2).
  2 $$ {\mathrm{Water}}\;{\mathrm{Absorption}}\left( \% \right) = 100 \times \left( {{\mathrm{W}}_{1} - {\mathrm{W}}_{2} } \right)/{\mathrm{W}}_{2} $$
- Apparent porosity (AP, %): Relates the volume of open pores to the total exterior volume of the specimen as per Eq. (3).
  3 $$ {\mathrm{Apparent}}\;{\mathrm{Porosity}}\left( \% \right) = 100 \times \left( {{\mathrm{W}}_{1} - {\mathrm{W}}_{2} } \right)/{\mathrm{V}}_{1} $$
- Bulk density (ρb, kg/m^3^): Determines the mass per unit overall volume (including both solid phases and pore voids) as per Eq. (4).
  4 $$ {\mathrm{Bulk}}\;{\mathrm{Density}} = {\mathrm{Mass}}\;{\mathrm{of}}\;{\mathrm{fired}}\;{\mathrm{bricks}}/{\mathrm{Volume}}\;{\mathrm{of}}\;{\mathrm{fired}}\;{\mathrm{bricks}} $$

where W_1_ is the mass of the wet, surface-dry saturated brick (kg), W_2_ is the dry mass of the fired brick (kg), and V_1_ is the total geometric volume of the fired specimen (m^3^). Cold water immersion testing was similarly implemented as boiled water test to determine standard structural absorption ratings.

#### Mechanical properties: compressive strength

Uniaxial compressive strength tests were performed on the 50 mm cubic specimens in accordance with ISO 9652-4^36^. The fired brick cubes were positioned within a calibrated universal testing machine (UTM) and subjected to a continuous, uniform perpendicular load until structural failure manifested. The maximum peak load sustained by the specimen was recorded. The ultimate compressive strength was calculated using Eq. (5).

5 $$ {\mathrm{Compressive}}\;{\mathrm{strength}}\;\left( {{\mathrm{MPa}}} \right) = {\mathrm{Load}}\;{\mathrm{at}}\;{\mathrm{failure}}\;\left( {\mathrm{N}} \right)/{\mathrm{cross-sectional}}\;{\mathrm{area}}\;{\mathrm{of}}\;{\mathrm{the}}\;{\mathrm{loaded}}\;{\mathrm{face}}\;\left( {{\mathrm{mm}}^{2} } \right) $$

#### Thermal properties: thermal conductivity and specific heat

Thermal performance metrics were characterized at ambient temperatures for specimens fired at 900 °C using a commercial KD2 Pro Thermal Properties Analyzer. The measurement process adhered strictly to the ASTM D5334 standard protocol, utilizing the transient line heat source technique. This non-steady-state approach digitally records the temperature response of the brick matrix to a localized thermal pulse over a short time interval. Alongside thermal conductivity (λ, W/m K), the specific heat (Cv, MJ/m^3^ K) of the 900 °C fired composite specimens was quantified to comprehensively model the thermal insulation efficiency and heat storage capacity of the developed eco-bricks.

## Results and discussion

All experimental test results are summarized in Table 2, while Table 3 summarized the p-values form two-way ANOVA analysis for OPW content and firing temperature. All individual p-values are much less than 0.05 which indicates that both OPW content and firing temperature have significant impact on the brick properties. Also, the interaction p-values are less than 0.05 (which indicated significant dependency) for all properties except apparent porosity, bulk density and linear Firing Shrinkage.

**Table 2:** Summary for all experimental test results.

	Orange peels %				
	0.0%	2.5%	5.0%	7.5%	10.0%
700 °C					
Apparent porosity (%)	30.28 ± 5.9%	31.26 ± 3.4%	32.42 ± 5.7%	35.6 ± 6.1%	38.87 ± 5.3%
Compressive strength (MPa)	11.38 ± 0.8%	9.75 ± 2.1%	8 ± 3.8%	6.79 ± 4.6%	6 ± 4.3%
Cold water absorption (%)	16.43 ± 3.1%	20.09 ± 4.5%	24.79 ± 4.9%	29.8 ± 2.5%	34.69 ± 4.1%
Boiling water absorption (%)	18.33 ± 3.4%	22.65 ± 2.7%	27.68 ± 2.4%	34.27 ± 5%	42.55 ± 1.9%
Bulk density (kg/m3)	1829 ± 0.8%	1487 ± 2.4%	1306 ± 1.3%	1210 ± 0.9%	1150 ± 2%
Linear firing shrinkage (%)	0.32 ± 5.4%	1.95 ± 3.5%	2.57 ± 5.4%	2.67 ± 4.7%	5.24 ± 4.4%
800 °C					
Apparent porosity (%)	28.61 ± 7.6%	29.57 ± 7.8%	30.5 ± 6.3%	33.4 ± 4.2%	37.01 ± 6.3%
Compressive strength (MPa)	14.88 ± 4.3%	13 ± 3.7%	11.03 ± 1.9%	9.35 ± 0.3%	7.56 ± 2.6%
Cold water absorption (%)	14.49 ± 1.5%	17.51 ± 6.6%	21 ± 3.9%	25 ± 6.4%	29.61 ± 6.8%
Boiling water absorption (%)	17.34 ± 0.5%	21.45 ± 2.7%	25.46 ± 0.8%	30 ± 1%	37.83 ± 4.4%
Bulk density (kg/m3)	1874 ± 2.7%	1599 ± 2.8%	1409 ± 1.2%	1300 ± 2.2%	1236 ± 1.4%
Linear firing shrinkage (%)	1.1 ± 2%	2.85 ± 5.6%	3 ± 4.9%	3.79 ± 4.8%	6.27 ± 5.9%
900 °C					
Apparent porosity (%)	26.6 ± 3.1%	27.07 ± 4.9%	27.6 ± 2.4%	29.98 ± 3.7%	32.88 ± 2.1%
Compressive strength (MPa)	20.76 ± 3.7%	19 ± 5.3%	16.28 ± 2.1%	13.27 ± 3.4%	10.68 ± 4.6%
Cold water absorption (%)	13.9 ± 3.8%	15.02 ± 4.1%	17.57 ± 2%	20.28 ± 1.8%	23.58 ± 0.8%
Boiling water absorption (%)	16.01 ± 2.8%	18.37 ± 2.7%	21 ± 2%	24.34 ± 1.2%	30.67 ± 2.1%
Bulk density (kg/m3)	1922 ± 1.9%	1646 ± 1.9%	1489 ± 0.8%	1400 ± 1.5%	1313 ± 1.7%
Linear firing shrinkage (%)	1.9 ± 1.7%	3.77 ± 5.6%	3.98 ± 3%	4.56 ± 4.6%	7.07 ± 3.7%
Thermal conductivity (W/m K)	0.8 ± 1.6%	0.62 ± 0.8%	0.58 ± 4%	0.47 ± 2%	0.42 ± 3%
Specific heat (MJ/m^3^.K)	1.68 ± 3.9%	1.59 ± 2.3%	1.46 ± 3.6%	1.39 ± 3.5%	1.33 ± 2.3%
120 °C					
Linear drying shrinkage (%)	2.76 ± 4.1%	3.58 ± 6.5%	4.06 ± 6.7%	5.7 ± 5.3%	6.96 ± 5.2%

**Table 3:** Summary for two-way ANOVA analysis.

	*p*-value		
	OPW%	Firing temp	Interaction
Apparent porosity (%)	1.7638E−06	1.92485E−08	0.989653331
Compressive strength (MPa)	7.39092E−26	7.3533E−23	1.55116E−06
Cold water absorption (%)	2.01285E−15	3.15812E−21	0.000110755
Boiling water absorption (%)	3.21786E−18	5.24468E−28	8.5219E−08
Bulk density (kg/m^3^)	1.073E−12	5.29928E−27	0.380832203
Linear firing shrinkage (%)	7.21035E−19	1.72996E−28	0.308677951

### Physical and linear expansion properties

The physical characteristics of the fired clay bricks incorporating varying weight percentages (0.0%, 2.5%, 5.0%, 7.5% and 10.0%) of (OPW) across different firing temperatures (700, 800 and 900 °C) are systematically examined below.

#### Bulk density

Bulk density is a fundamental physical property that heavily dictates the dead weight of construction components and directly influences mechanical performance. As the incorporation level of (OPW) powder increases from 0.0 to 10.0%, a distinct and consistent downward trend in bulk density is observed across all three sintering temperatures (700, 800 and 900 °C). In accordance with ASTM C55-22^37^, bricks with a dry density exceeding 2000 kg/m^3^ are classified as normal-weight. Accordingly, the reference mix and low-OPW specimens demonstrated dry densities within this range and can be categorized as normal-weight bricks. With further OPW incorporation, the dry density shifted into the 1680–2000 kg/m^3^ range, corresponding to medium-weight bricks, while specimens containing the highest OPW content exhibited dry densities below 1680 kg/m^3^, thereby meeting the criteria for lightweight bricks. The decrease in density is related to the lightweight property of OPW, which has a significantly lower specific gravity than clay. The decrease in density is related to the lightweight property of OPW, which has a significantly lower specific gravity (0.96 g/cm^3^) than clay (2.78 g/cm^3^), due to its high porous nature. The increase in the amount of OPW addition causes a reduction in the brick density. This reduction is fundamentally attributed to the thermal volatilization and complete combustion of the organic biomass additives during the firing stage. The elimination of the plant tissue leaves behind an interconnected network of micro-voids and artificial pores within the clay matrix. This behavior mirrors trends noted in literature for similar fruit-derived and agricultural biomass additives, such as pomegranate peel (which achieves up to a 26% reduction in bulk density) and wine industry wastes. Furthermore, a decrease in silica content aids in increasing porosity, which subsequently leads to lower density and thermal conductivity.

**Fig. 6:**
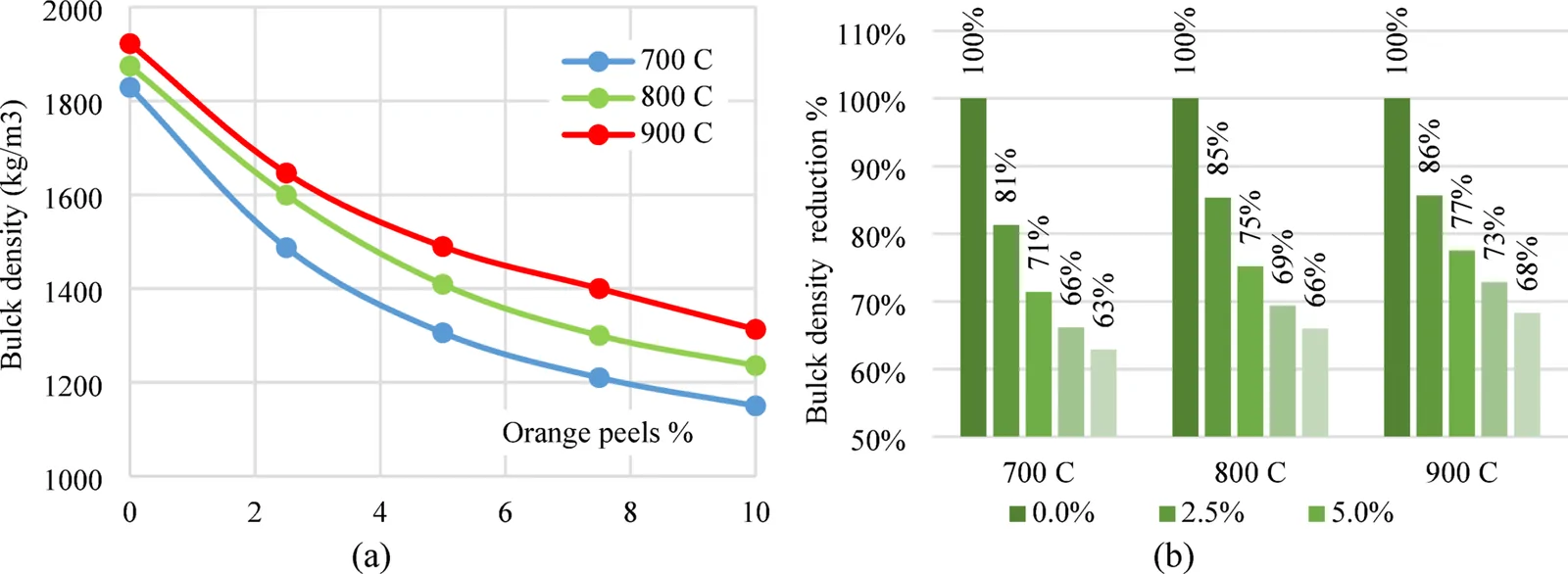
(**a**) Impact of orange peels content of the bulk density, (**b**) Bulk density reduction.

Concurrently, elevating the firing temperature from (700 to 900 °C) induces a slight increase in the bulk density for any given additive concentration. This phenomenon is driven by the progressive vitrification of the clay minerals, prompting structural consolidation, pore shrinkage, and the partial elimination of open micro-voids as the liquid phase fills the capillary pores. See Fig. 6a,b. According to the obtained bulk density, the brick can be considered light weight when it achieves a density of less than 1680 kg/m^3^ according to ASTM C90^38^.

#### Apparent porosity and water absorption (cold and boiled)

**Fig. 7:**
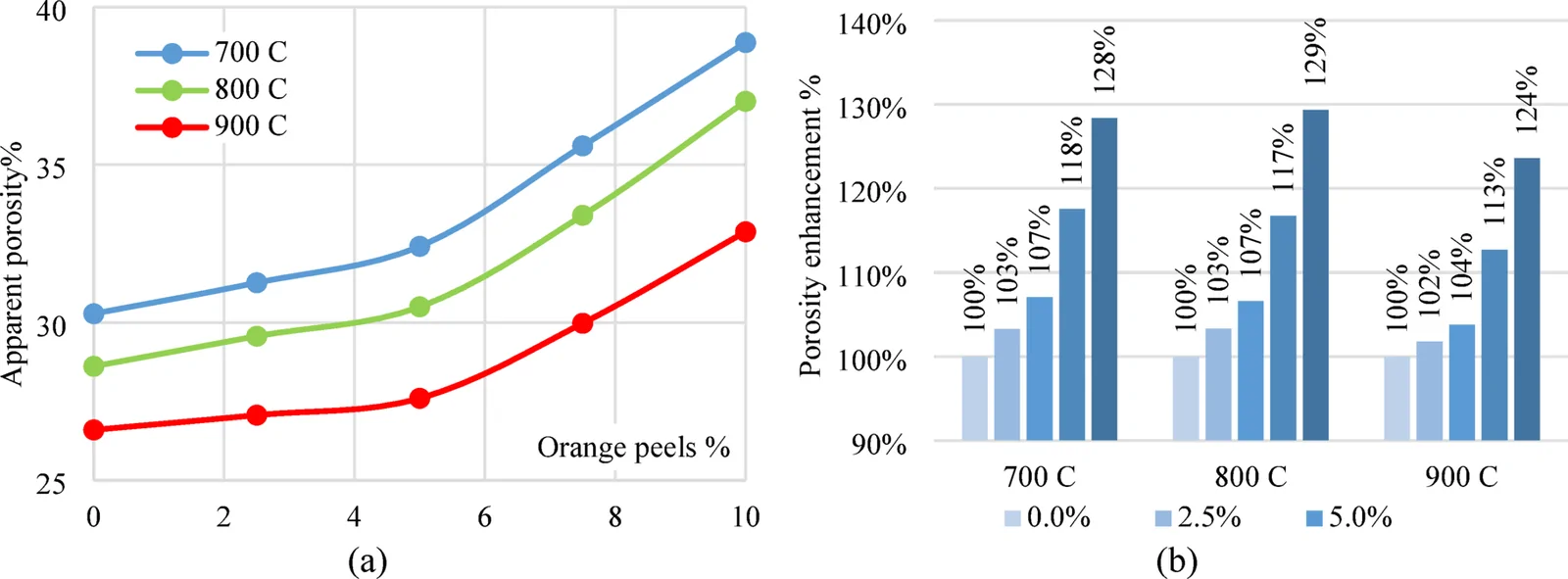
(**a**) Impact of orange peels content of the apparent porosity, (**b**) porosity enhancement.

Apparent porosity and water absorption capacity (evaluated via both cold and boiled immersion techniques) share an explicitly proportional relationship with the content of the pore-forming agent. The highest Porosity was seen in OPW-10. There is a close relationship between the bulk density and porosity of brick as shown in Fig. 7a,b ,it is clearly seen that while density decreases, porosity increases for all firing temperatures.

The apparent porosity of fired bricks varies inversely with firing temperature and increases with OPW addition due to burning and solidification of organic-waste.

Across all experimental batches, the introduction of higher (OPW) concentrations generates an increase in both apparent porosity and water absorption.

**Fig. 8:**
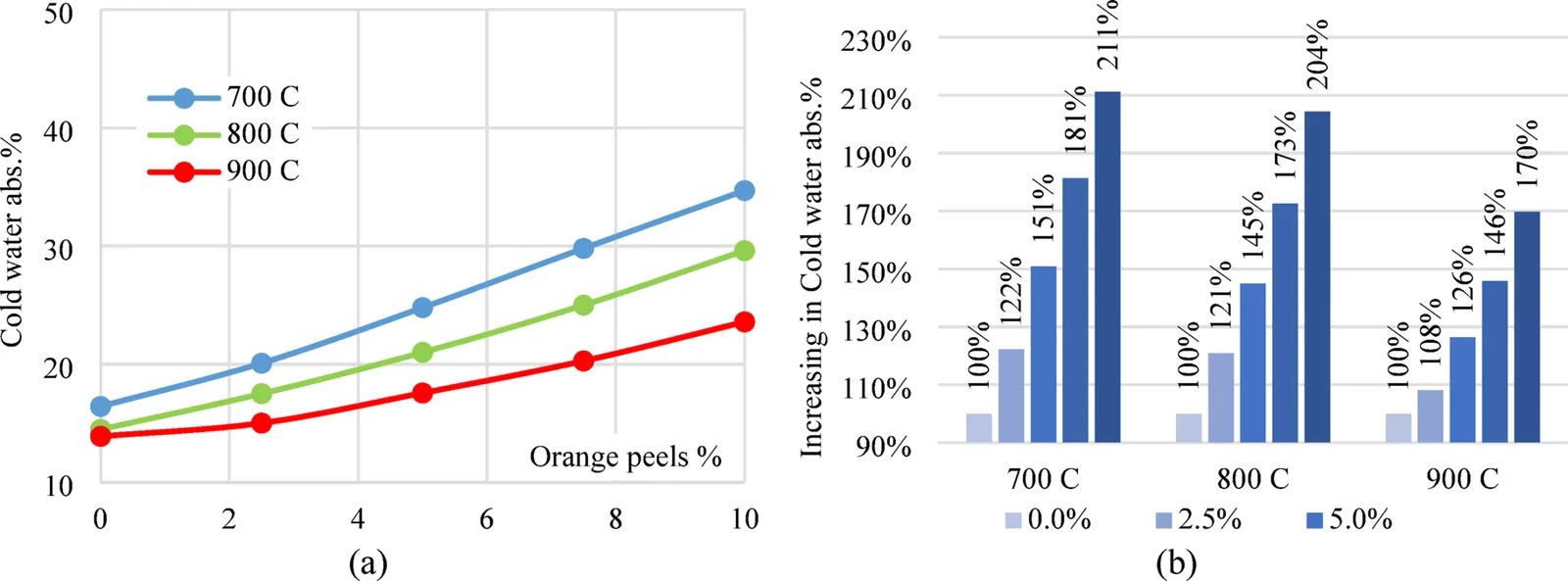
(**a**) Impact of orange peels content of the cold water absorption, (**a**) cold water absorption enhancement.

The addition of OPW (in its varying percentages) had a greater effect on the hydric behavior of the bricks than changes in the firing temperature. Figure 8a,b clearly shows that when the amount of OPW is increased, their water absorption capacity also increases. This is manifested in the decomposing of organic compound and produce CO_2_ gas and water vapor; these gases will move out, producing capillaries in the sample bricks. With increasing the OPW, a lower amount of clay will be used; therefore, a relatively low amount of liquid phase will be produced during the fusion. Hence a relatively larger amount of the open pores will remain. This can explain the increase in porosity and water absorption.

During the sintering process, the combustion of the fibrous and carbonaceous fractions inherent to the (OPW) architecture expands the internal void volume. These findings correlate strongly with the SEM images presented in Section “Microstructural analysis (SEM commentaries)”. For instance, at a low additive concentration 2.5%, the microstructural voids remain comparatively isolated and minor (5.58–15.33 μm). However, when the concentration is scaled to 10%, the burning out of the biomass yields vast, highly connected macro-pores ranging between 22.27 and 43.98 μm.

This progressive evolution into a more open, porous network explains the linear spike in water absorption. Higher firing temperatures (900 °C) partially mitigate this excessive porosity through enhanced solid-state diffusion and glassy phase formations, which seal off portions of the capillary channels.

**Fig. 9:**
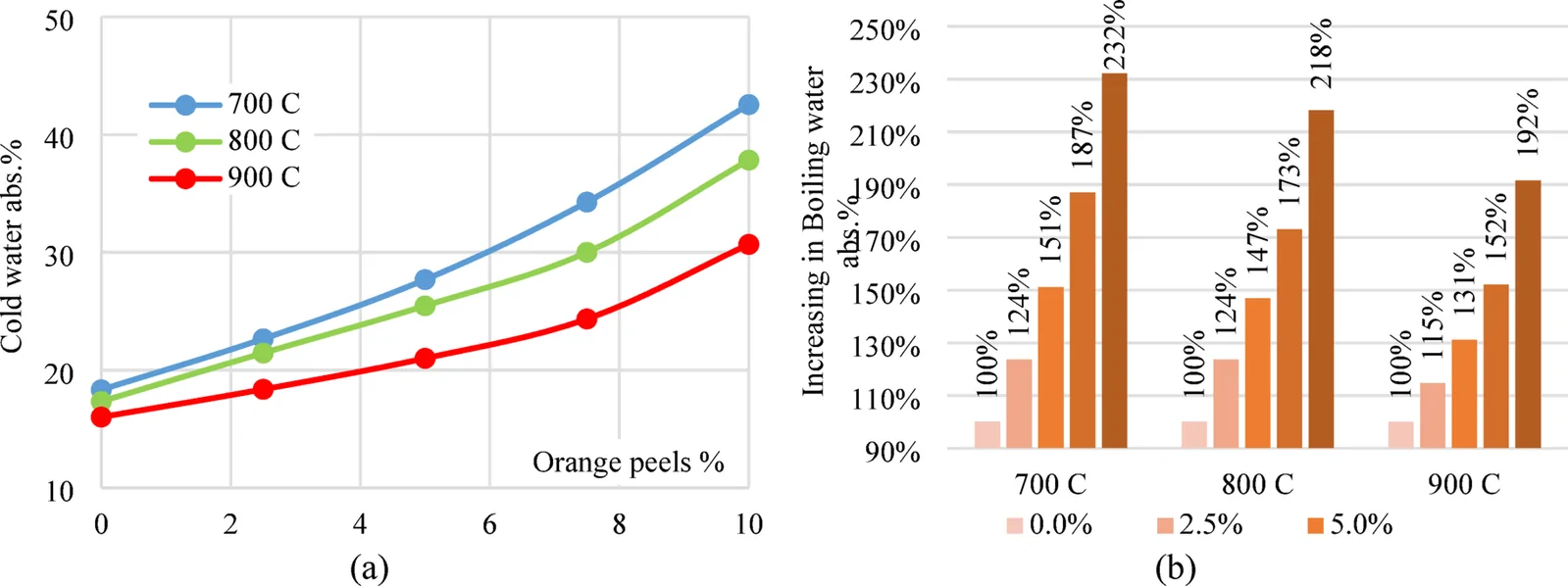
(**a**) Impact of orange peels content of the boiling water absorption, (**b**) increasing boiling water absorption.

Figure 9a,b shows that there is a general increase in boiling water absorption values with increased waste addition It is clear from the figure that the values of BWA starts increasing on firing when a substitution level of 10% is reached. This can be interpreted in light of the chemical composition of the OPW which reveals that the percent fluxing alkali oxides (Na_2_O+K_2_O) is about 0.27%.It is known that the presence of alkali oxides, unparticular, causes a serious drop in the melting temperatures of clay based ceramic bodies, which explains the observed increase in water absorption associated with increased waste addition.

#### Linear drying shrinkage (LDS) and linear firing shrinkage (LFS)

Linear shrinkage parameters must be carefully regulated to prevent internal micro-cracking and macro-structural warping during industrial brick processing.

**Fig. 10:**
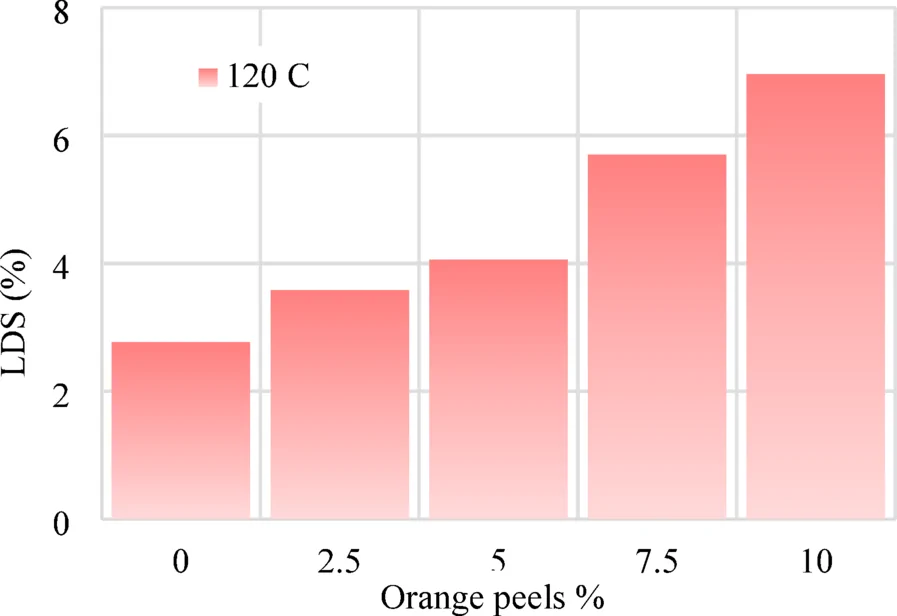
Impact of orange peels content of the linear drying shrinkage (LDS) at 120 °C.

**Fig. 11:**
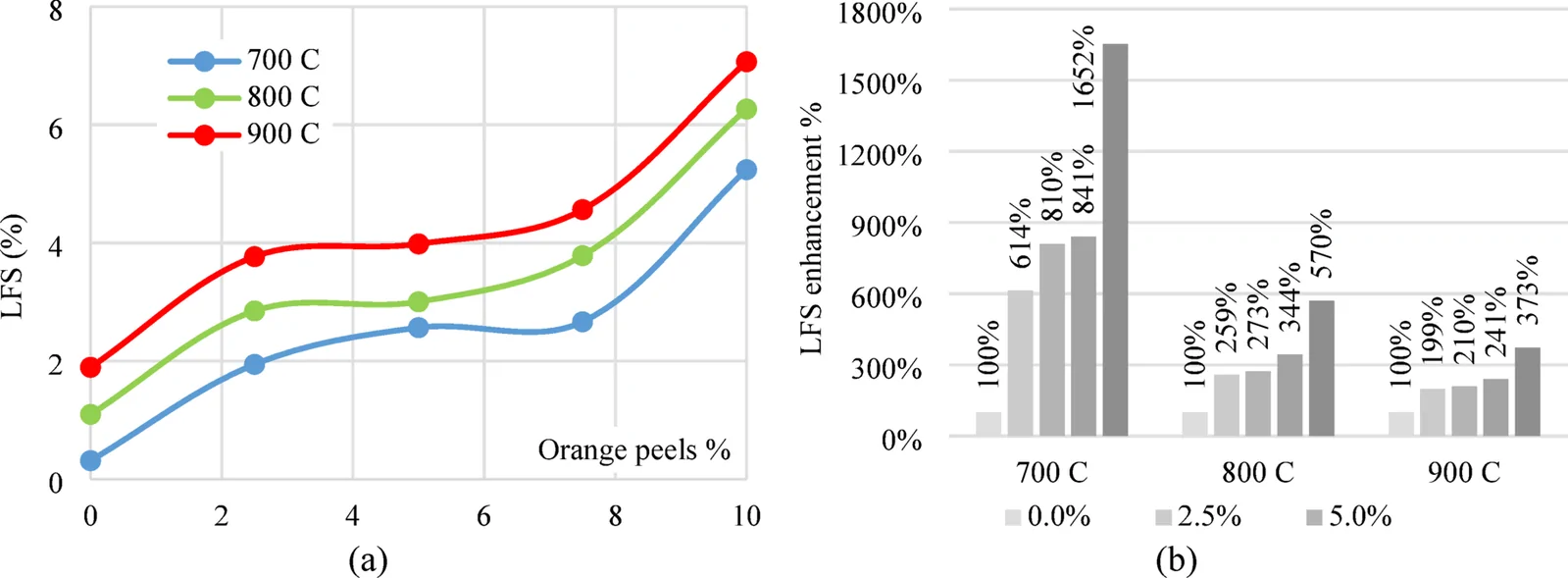
(**a**) Impact of orange peels content of the linear firing shrinkage (LFS), (**a**) (LFS) enhancement.

- *Linear drying shrinkage (LDS)*: The Linear Drying Shrinkage of bricks can be significantly enhanced when OPW is used as a substitute for clay and then dried. Figure 10 shows a slight decrease or remains highly stable as the biomass substitution increases. Replacing plastic clay matter with non-plastic, fibrous (OPW) particles diminishes the volume of water bound within the clay sheets, thereby limiting capillary contraction during the low-temperature moisture-removal stage.
- *Linear firing shrinkage (LFS)*: Fig. 11a,b illustrates the variation in LFS of bricks incorporating different proportions of OPW. LFS displays a progressive increase with both ascending biomass content and rising temperature. The inclusion of biomass leaves behind unfilled void volumes that undergo accelerated structural collapse and localized contraction during the high-temperature vitrification window (900 °C), prompting greater macroscopic dimensional changes.

### Mechanical Performance (compressive strength)

**Fig. 12:**
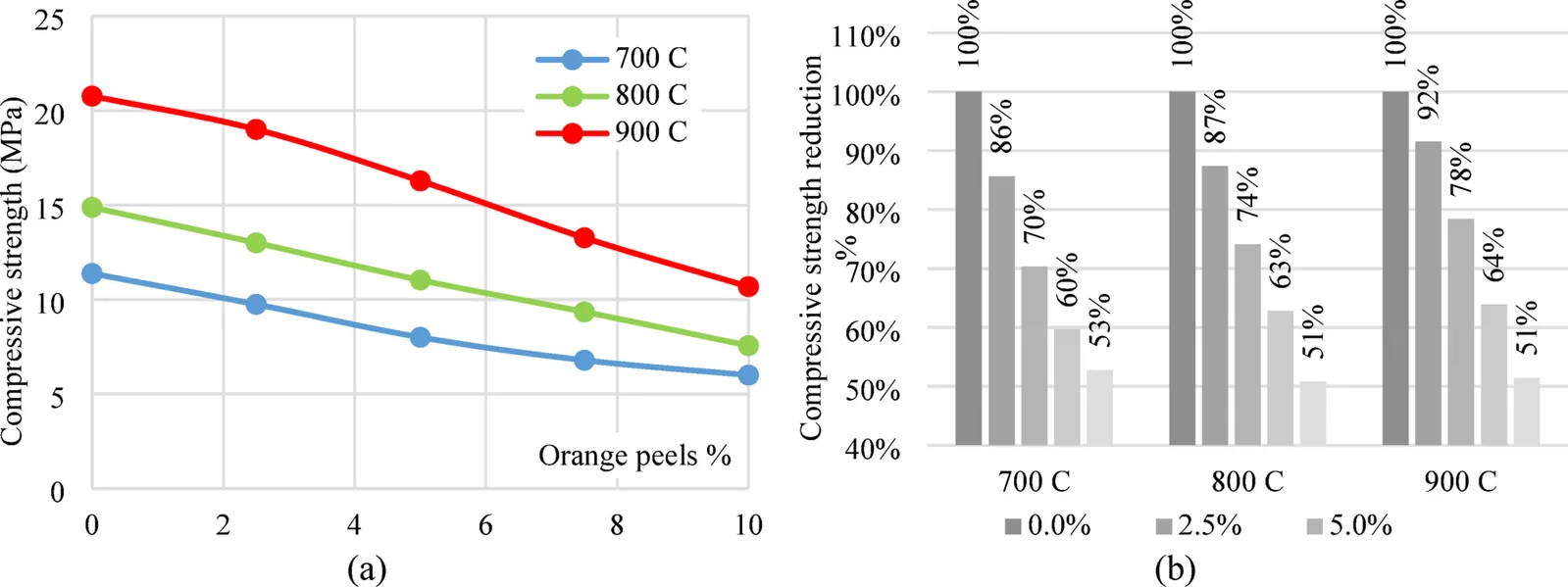
(**a**) Impact of orange peels content of the compressive strength, (**b**) reduction in compressive strength.

Compressive strength stands as the primary structural threshold validating the real-world deployment of alternative brick units. Experimental data reveals a classic inverse correlation between (OPW) concentration and compressive strength. As the additive content steps up from 0.0% to 10%, a distinct drop in mechanical resistance occurs across all firing temperatures (700–900 °C). See Fig. 12a,b.

This deterioration is explicitly structural: compressive strength is fundamentally governed by the effective cross-sectional area of the solid matrix capable of bearing external load. The creation of substantial micro- and macro-pores following biomass combustion substantially reduces this load-bearing area and concentrates localized stresses around the pore perimeters. This pattern is highly consistent with traditional biomass-blended bricks; for example, potato peel powder has been reported to drop compressive strength by up to 88% at a 7% substitution rate. Mandal et al.^39^ discovered that fired bricks with 10% sawdust had strengths of 0.05, 0.53, and 4.0 MPa at 1000, 1100, and 1200 °C. At higher firing temperatures, OPW reacts with clay particles, forming new crystalline phases in the burned body. Finally, the strength of the brick construction grows. Still, sufficient strength for structural applications was acquired according to the regulations specified above. According to TS EN 771-1^40^, Compressive strength should not less than about 7 MPa. Similar findings were discovered by Gencel et al.^27^, indicating that the addition of concrete waste reduced the mechanical strength of the brick, which can be attributed to the chemical and physical structure of the concrete waste. The reference sample had the maximum compressive strength, which corresponded to its largest unit weight; however, these samples also had the lowest apparent porosity and water absorption. According to disaster regulation^41^, the minimum strength for building bricks is 5 MPa and the necessary compressive strength for horizontal perforated brick in TS EN 771-1 is between 2.5 and 7.5 MPa^42^. In this study, all of the bricks fired from (700 to 900 °C) were provided the necessary compressive strength. Reducing the compressive strength of the bricks, which may be due to lack of SiO_2_, and thus greater pore volume^43^.

Importantly, the thermal treatment acts as a major compensating factor. Bricks fired at (900 °C) display vastly superior compressive strengths compared to those fired at (700 and 800 °C). Sintering at (900 °C) triggers the de-hydroxylation of clay minerals and initiates vitrification, forming a durable glassy phase that reinforces the remaining skeletal structure. Consequently, while the 10.0% (OPW) sample exhibits the lowest mechanical strength, the samples containing 2.5–5.0% biomass fired at (900 °C) comfortably exceed the minimum regulatory structural thresholds designated by international standards for non-load-bearing partition walls and lightweight thermal envelopes. This finding corroborates the previously published researches^44,45^.

### Thermal characteristics (thermal conductivity and specific heat)

A primary objective of incorporating (OPW) is to optimize the thermal insulation properties of the building envelope, thereby lowering operational energy consumption in buildings. Thermal conductivity and specific heat were characterized at the optimal sintering temperature of (900 °C) as shown in Table 4.

**Table 4:** Ranking of thermal conductivity and specific heat for different Temperatures.

(OPW) content (wt%)	Thermal conductivity (W/m K)	Specific heat (MJ/m^3^ K)
0.0% (Control)	Highest	Lowest
2.5%	Moderate reduction	Incremental rise
5.0%	Substantial decrease	Noticeable increase
7.5%	Advanced decay	High value
10.0%	Lowest value	Highest value

**Fig. 13:**
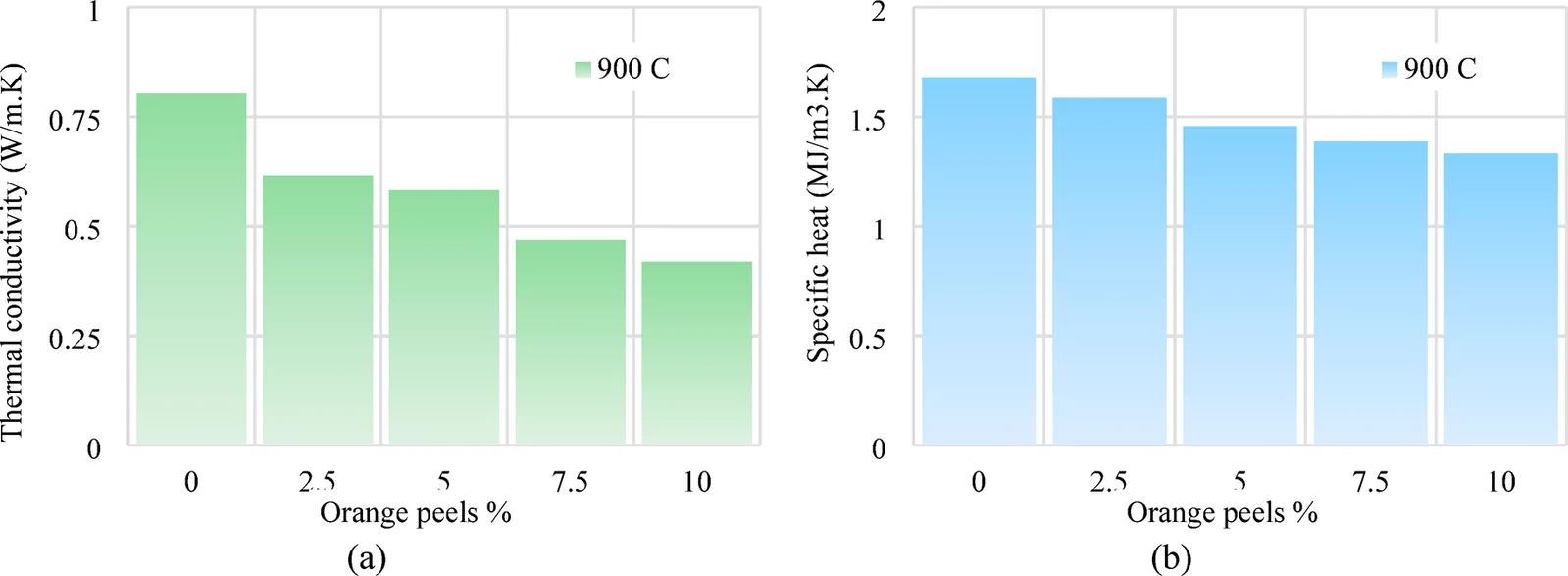
Impact of orange peels content on (**a**) thermal conductivity, and (**b**) specific heat.

The data in Fig. 13a,b reveals a dramatic reduction in thermal conductivity as the biomass content scales toward 10%. This improvement is directly tied to the induced porosity. Air trapped within the newly created micro-pores possesses an exceptionally low thermal conductivity (≈ 0.026 W/m K), effectively turning the macro-voids into thermal barriers that inhibit conductive heat transfer across the brick bulk. This aligns closely with previous literature where pomegranate peel achieved thermal conductivity reductions of 62% to 97.7%, and mushroom or olive pomace waste produced similarly enhanced insulation profiles.

Simultaneously, the specific heat capacity scales upward with higher biomass addition. Porous structures slow down thermal response times, increasing the overall thermal inertia of the brick material. This combination of reduced thermal conductivity and elevated specific heat makes these green bricks highly effective for hot-arid and temperate climates, where stabilizing indoor temperatures and reducing cooling loads are key design priorities.

### Microstructural analysis (SEM commentaries)

**Fig. 14:**
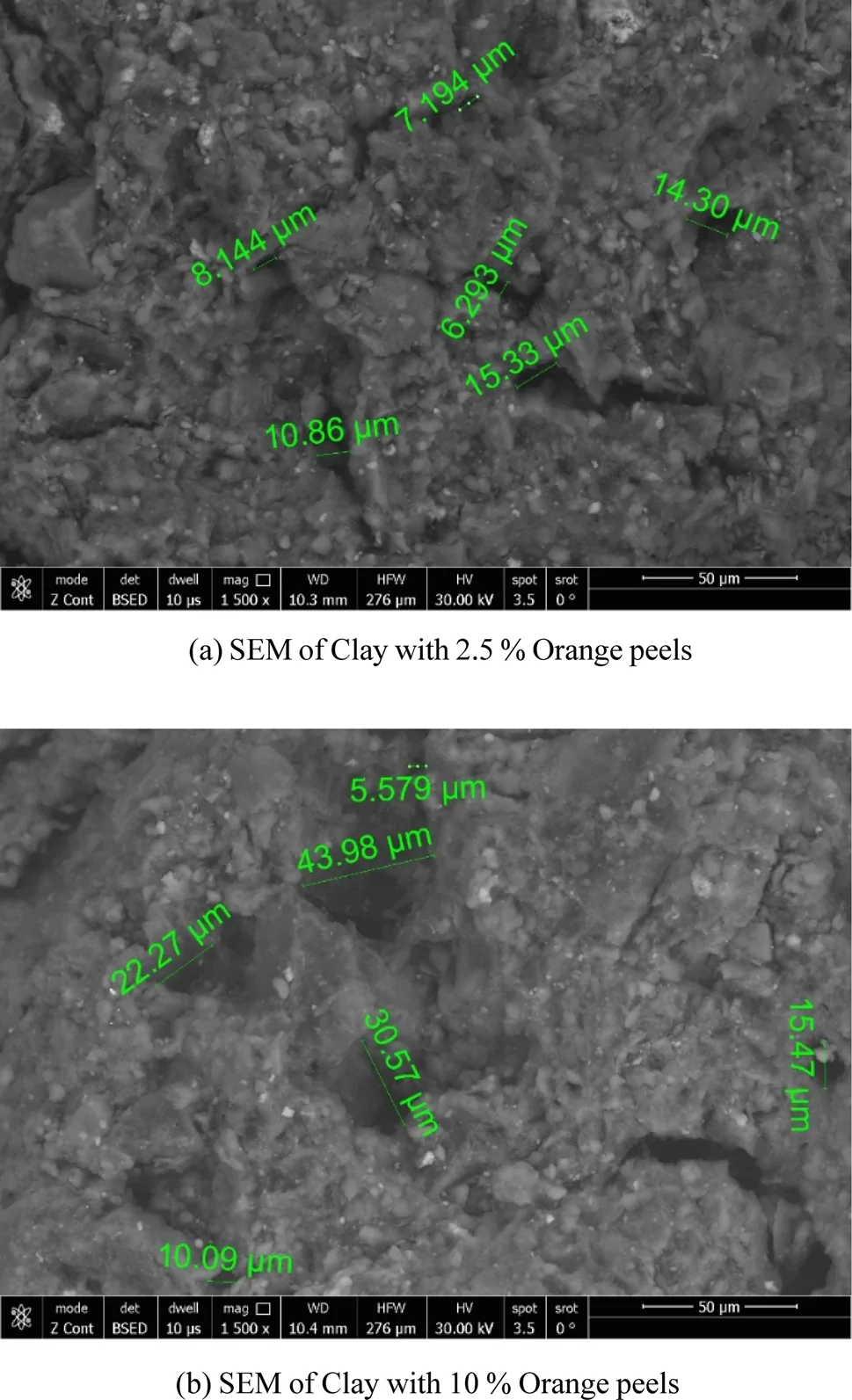
SEM at 900 °C for (**a**) clay mixed with 2.5, (**b**) clay mixed 10% Orange peels.

To unveil the physical mechanisms responsible for these altered macro-properties, scanning electron microscopy (SEM) was conducted on representative samples containing 2.5% and 10.0% orange-derived biomass. See Fig. 14a,b.

#### Microstructure of clay with 2.5% biomass

The SEM micrograph of the clay sample blended with 2.5% biomass captures a highly consolidated, dense, and uniform structural matrix. At this low dosing level, the clay phase remains dominant and continuous. The internal void features are sparse and small, with explicitly mapped pore sizes tracking at 6.293 μm, 7.194 μm, 8.144 μm, and 10.86 μm. A couple of elongated voids measuring 14.30 μm and 15.33 μm are visible, corresponding to the volatile release sites of isolated organic fractions. Because the solid clay matrix remains largely uninterrupted, this structural configuration explains the high bulk density and superior compressive strength observed at lower substitution values.

#### Microstructure of clay with 10.0% biomass

Elevating the biomass incorporation to 10% radically transforms the structural topography. The SEM image shows a highly fractured, cavernous, and open microstructural matrix plagued by extensive pore formation. The tiny micro-pores seen in the 2.5% sample have coalesced into large, irregular macro-structural cavities.

While a few minor residual voids persist (5.579 μm and 10.09 μm), the microstructure is dominated by vast cavities measuring 15.47 μm, 22.27 μm, 30.57 μm, and up to 43.98 μm. These prominent macro-pores are the direct footprint of the completely burned-out organic mass. This image provides direct microstructural evidence explaining the stark drop in bulk density, the sharp increase in apparent porosity/water absorption, and the significantly enhanced thermal and acoustic insulation properties found at higher substitution rates.

### Elemental composition and mineralogical insights (EDX analysis)

**Fig. 15:**
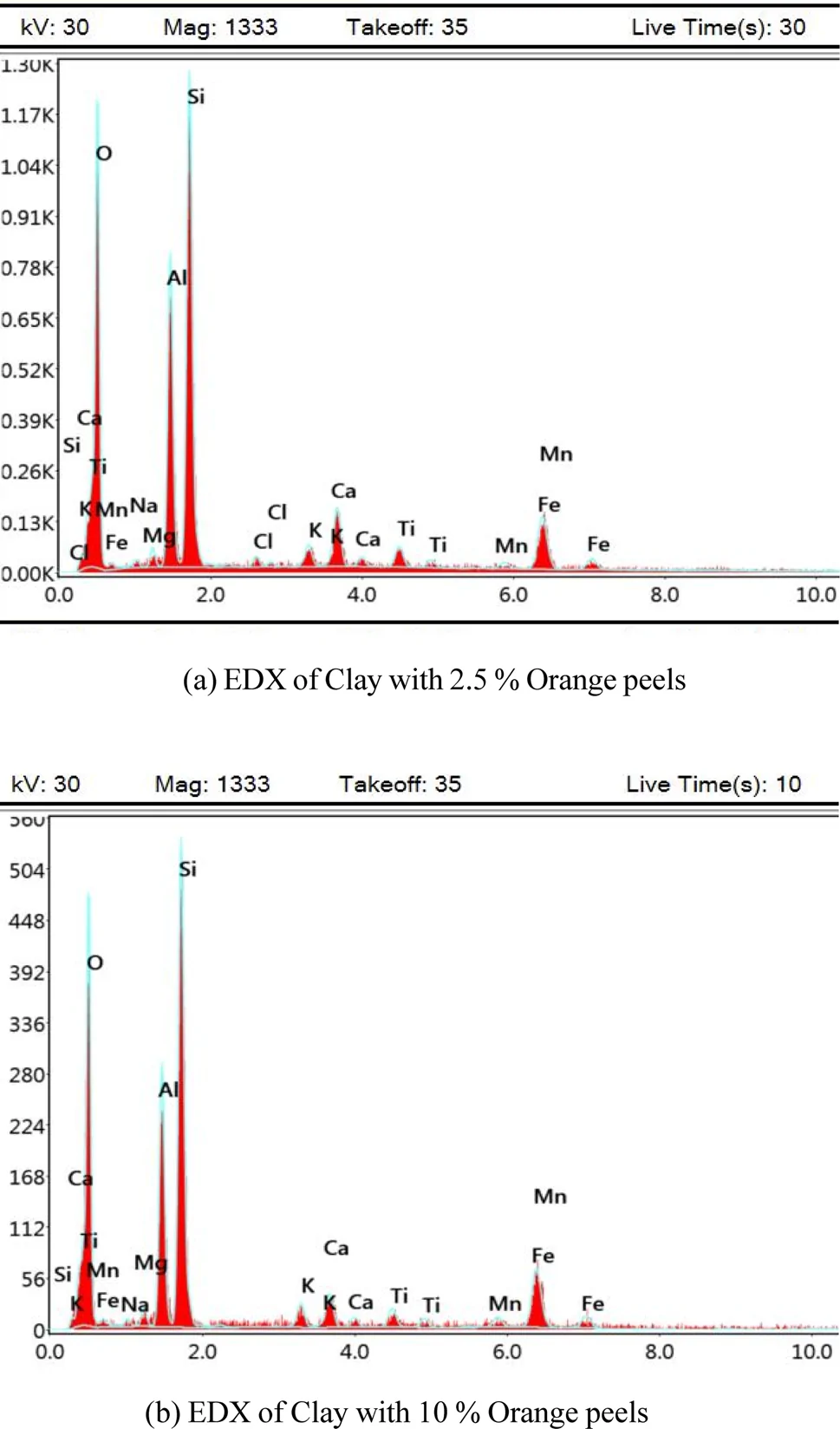
EDX at 900 °C of Clay mixed with (**a**) 2.5 & (**b**) 10% Orange peels.

Energy-dispersive X-ray spectroscopy (EDX) was used to tracking elemental modifications across the brick profiles and evaluate the chemical purity and binding attributes of the clay-biomass mixtures. See Fig. 15a,b.

#### EDX analysis of 2.5% biomass mix

The EDX spectrum and corresponding quantitative data for the 2.5% biomass sample reflect a classic aluminosilicate clay signature. The dominant elements are Oxygen (O: 53.8%), Silicon (Si: 20.82%), and Aluminum (Al: 13.48%), confirming a stable quartz (SiO_2_) and kaolinite/aluminosilicate mineral frame.

Fluxing oxides and auxiliary elements are present in typical proportions: Iron (Fe: 4.17%, which imparts the classic red color upon firing), Calcium (Ca: 2.92%), Magnesium (Mg: 1.12%), Titanium (Ti: 1.12%), Potassium (K: 0.95%), and Sodium (Na: 0.7%). Trace levels of Chlorine (Cl: 0.49%) and Manganese (Mn: 0.44%) are also recorded. The low error percentages (e.g., 5.31% for Fe, 7.18% for Si) validate the high precision of this elemental profile.

#### EDX analysis of 10.0% biomass mix

When shifting focus to the 10% biomass spectrum, the elemental distribution remains fundamentally rooted in the aluminosilicate matrix but shows subtle, distinct shifts. O stands at 52.52%, Si slightly increases to 22.61%, and Al sits at 12.64%. Crucially, alkaline and earth-alkaline fluxing elements shift slightly: Potassium increases to 1.17% and Sodium to 0.85%, reflecting the mineral ash remnants (rich in potassium and soluble salts) left behind by the organic orange peel tissue. Iron concentrations increase to 5.18%, while Calcium drops slightly to 1.85%. Magnesium (Mg: 1.3%) and Manganese (Mn: 0.79%) complete the profile.

### Optimization matrix and comparative analysis

To determine the optimal concentration of (OPW), the competing requirements of physical durability, mechanical strength, and thermo-acoustic insulation must be carefully balanced.

While a 10% addition maximizes thermal insulation and sound deadening, it triggers an extensive drop in compressive strength and a sharp increase in water absorption, which could jeopardize long-term durability under severe environmental exposure. Conversely, the 2.5% mix preserves excellent structural strength but yields only minor improvements in insulation performance.

Therefore, the 5.0% to 10% (OPW) incorporation range, combined with a firing temperature of (900 °C), represents the optimal design formulation. This specific configuration produces a lightweight brick that successfully capitalizes on biomass-induced pore formation to deliver excellent thermal insulation, while safely maintaining its structural integrity above the mandatory thresholds required for modern building partition walls.

### Comparative synthesis with previous literature

To contextualize the performance of the (OPW)-enhanced bricks, Table 5 compares the properties of the optimized samples from this study against other prominent agro-waste brick alternatives documented in the literature.

**Table 5:** Comparative performance of various agro-waste-enhanced fired clay bricks.

Additive type	Optimal content (%)	Firing temp (°C)	Prominent physical/mechanical effects	Thermal & acoustic impacts	Key reference
Potato peel powder	7%	–	Compressive strength reduced by up to 88%; increased porosity.	Enhanced sound insulation.	Ghorbani et al.^10^
Pomegranate peel	10–15%	900–1000	Bulk density reduced by up to 26%.	Thermal conductivity decreased by 62–97.7%.	Ragab et al.^16^
Fruit seeds & wine lees	5%	–	Weight reduction of 3–13%; maintained flexural strength (21–23 MPa).	Suitable insulation for partition walls.	Taurino et al.^14^
Mushroom waste	15%	–	Bulk density reduced by 26%; satisfied structural criteria.	Thermal conductivity decreased by 62%.	Ahmed et al.^15^
Orange peels & coconut	10–60%	–	Lightweight, shock-absorbing bricks; satisfied ASTM/BIS standards.	Confirmed internal pores via SEM.	Arshad^18^
Orange peels waste	2.5–5%	900	Balanced density drop with structural integrity; dense matrix at 2.5% vs. macro-pores up to 44 μm at 10%.	Significant thermal conductivity drop; optimized acoustic attenuation.	Present study

## Conclusions

The present study systematically investigated the incorporation of (OPW) as an eco-friendly pore-forming agent in fired clay bricks across different sintering temperatures (70, 800 and 900 °C). Based on the experimental findings, microstructural evaluations, and elemental analyses, the following conclusions can be drawn:

- Physical and density enhancements: The addition of (OPW) successfully produced lightweight bricks. Increasing the biomass content from 0.0% to10.0% yielded a progressive reduction in bulk density due to the thermal combustion of organic matter during firing. This reduction was accompanied by a linear increase in apparent porosity and water absorption values.
- Mechanical viability: Although an increase in biomass content caused a corresponding reduction in compressive strength due to the formation of structural macro-pores, sintering at (900 °C) significantly mitigated this loss. Bricks containing up to 5.0–10% (OPW) fired at (900 °C) safely satisfied the minimum regulatory mechanical benchmarks required for non-load-bearing structural applications.
- Improved thermo-acoustic insulation: At (900 °C), the developed bricks exhibited superior thermal and acoustic performance. The thermal conductivity dropped substantially as a result of the entrapped air within the newly created micro-voids, while the specific heat capacity increased. Concurrently, the tortuous pore network significantly enhanced acoustic damping, making these bricks highly effective for noise-reducing interior partition walls.
- Microstructural and elemental validation: SEM analysis confirmed the evolution from isolated micro-pores (5.58–15.33 μm) at a 2.5% dosing level to a highly interconnected network of macro-cavities (up to 43.98 μm) at a 10.0% dosing level.
- Optimal formulation: The formulation incorporating 5.0–7.5% (OPW) sintered at (900 °C) offers the optimal balance between lightweight properties, thermal insulation, acoustic performance, and structural integrity.

### Study limitations

While this research establishes the viability of using (OPW) to manufacture sustainable, energy-efficient bricks, certain limitations must be acknowledged:

- The laboratory-scale manufacturing process utilized manual mixing and small-scale kilns, which may introduce dimensional and structural variations compared to automated, industrial-scale extrusion and continuous tunneling kiln techniques.
- Long-term durability metrics—such as freeze-thaw resistance, efflorescence behavior, toxic heavy metal leaching, and chemical degradation under acidic or saline conditions—were not explicitly evaluated in this phase of work.
- Due to limited budget, advanced tests such as SEM, EDX, and XRD were conducted on selected samples only.

### Recommendations for future research

To build upon the insights gained from this study and facilitate the commercial adaptation of biomass-blended bricks, the following future research pathways are recommended:

- Investigate the performance of the optimized (OPW) bricks under rigorous weathering conditions, focusing on efflorescence, freeze-thaw cycles, and long-term structural stability under sustained mechanical loads.
- More advanced tests are required to understanding phase transformations and verifying vitrification such as SEM, EDX, XRD and TGA/DTG tests for samples with different OPW content and different firing temperatures.
- Life cycle assessment (LCA): Conduct a comprehensive LCA and economic feasibility study to precisely quantify carbon footprint reductions, energy savings during firing, and the overall commercial viability of managing agricultural waste streams through this circular economy approach.
- Alternative sintering technologies: Explore the integration of chemical fluxing agents or advanced low-temperature sintering aids to achieve the same degree of vitrification below (900 °C), further minimizing production energy demands.

## Data Availability

All data used are included in Table 2.
